# Implication of Nanoparticles to Combat Chronic Liver and Kidney Diseases: Progress and Perspectives

**DOI:** 10.3390/biom12101337

**Published:** 2022-09-21

**Authors:** Mariam Hashim, Huma Mujahid, Samina Hassan, Shanila Bukhari, Iram Anjum, Christophe Hano, Bilal Haider Abbasi, Sumaira Anjum

**Affiliations:** 1 Department of Biotechnology, Kinnaird College for Women, Jail Road, Lahore 54000, Pakistan; 2 Department of Biochemistry and Biotechnology, University of Veterinary and Animal Sciences, Lahore 54000, Pakistan; 3 Department of Botany, Kinnaird College for Women, Jail Road, Lahore 54000, Pakistan; 4 Department of Biological Chemistry, University of Orleans, Eure & Loir Campus, 28000 Chartres, France; 5 Department of Biotechnology, Quaid-i-Azam University, Islamabad 15320, Pakistan

**Keywords:** kidney, liver, toxicity, nanoparticles, anti-inflammatory, antioxidant, pro-oxidant

## Abstract

Liver and kidney diseases are the most frequently encountered problems around the globe. Damage to the liver and kidney may occur as a result of exposure to various drugs, chemicals, toxins, and pathogens, leading to severe disease conditions such as cirrhosis, fibrosis, hepatitis, acute kidney injury, and liver and renal failure. In this regard, the use of nanoparticles (NPs) such as silver nanoparticles (AgNPs), gold nanoparticles (AuNPs), and zinc oxide nanoparticles (ZnONPs) has emerged as a rapidly developing field of study in terms of safe delivery of various medications to target organs with minimal side effects. Due to their physical characteristics, NPs have inherent pharmacological effects, and an accidental buildup can have a significant impact on the structure and function of the liver and kidney. By suppressing the expression of the proinflammatory cytokines iNOS and COX-2, NPs are known to possess anti-inflammatory effects. Additionally, NPs have demonstrated their ability to operate as an antioxidant, squelching the generation of ROS caused by substances that cause oxidative stress. Finally, because of their pro-oxidant properties, they are also known to increase the level of ROS, which causes malignant liver and kidney cells to undergo apoptosis. As a result, NPs can be regarded as a double-edged sword whose inherent therapeutic benefits can be refined as we work to comprehend them in terms of their toxicity.

## 1. Liver and Renal Toxicity

**Figure 1:**
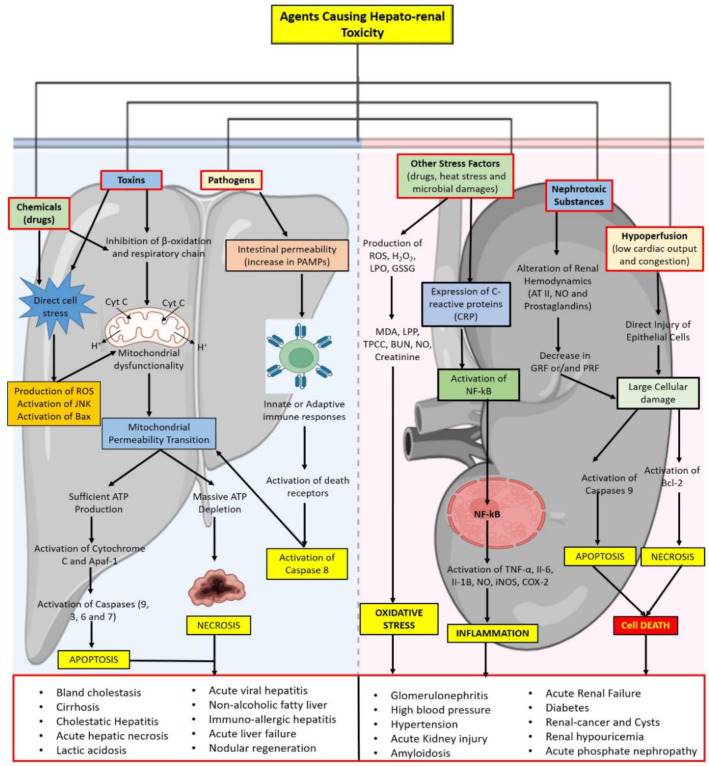
A phenomenological model of hepato-renal toxicity.

The liver is one of the largest organs in the human body and the primary focus of metabolic and excretory activity [1]. It is responsible for the detoxification and excretion of a wide range of endogenous and exogenous substances and any damage to it or impairment of its functioning can have serious consequences for one’s health [2]. Certain chemicals, drugs, food, and a variety of infections (bacterial, fungal, or viral) may cause liver diseases such as hepatitis, cirrhosis, jaundice, and liver cancer [3]. Liver diseases are among the major serious disorders resulting from acute and chronic hepatitis (inflammatory liver diseases), hepatosis (non-inflammatory liver disorders), and cirrhosis (a degenerative disorder leading to liver fibrosis). Other major causes of liver ailments may also include toxic drugs (certain antibiotics, chemotherapeutics, peroxidized oil, aflatoxin, carbon tetrachloride, acetaminophen, chlorinated hydrocarbons), excessive alcohol use, infections, and autoimmune disorders (Figure 1) [4,5]. The majority of hepatotoxic substances harm liver cells by causing lipid peroxidation and other oxidative damage. Viruses are thought to be responsible for 90 percent of acute hepatitis cases. Hepatitis B, A, C, D (delta agents), E, and G are the primary viral agents responsible for contributing to liver failure. Hepatitis B infection is the most common cause of chronic liver disease and liver cirrhosis. These viruses have also been linked to the development of primary liver cancer. In the Southeast Asian region, it is estimated that 14–16 million individuals are infected with the hepatitis B virus, with around 6% of the overall population carrying the virus [5]. Similarly, endogenous and exogenous chemicals are secreted mostly by the kidneys. The kidney filters all harmful substances and metabolites are eliminated through the urine. Chemicals that cause renal toxicity can cause acute renal failure either directly through a biotransformation process in the proximal tubular cells or indirectly by the creation of toxic metabolites in the liver that have nephrotoxic effects [6]. Because it filters a huge number of toxins that can accumulate in the renal tubules when a vast volume of blood flows through it, the kidney is extremely vulnerable to toxicants. This can then lead to systemic toxicity, which can impair bodily functions such as maintaining the fluid and electrolyte balance, decreasing the production of essential hormones, and impairing the body’s ability to expel wastes [7]. The primary pathologies contributing to kidney problems include oxidative stress, inflammation, apoptosis, and necrosis [8]. Two-thirds of chronic kidney disease cases are caused by diabetes, glomerulonephritis, high blood pressure, or hypertension. Along with this, acute renal failure also leads to nephrotoxicity, which is caused by nephrotoxic substances, drugs, and hypoperfusion [9].

The main agents causing hepato-renal toxicity are shown in Figure 1. In the case of hepatotoxicity, direct cell stresses induced through chemicals lead to the activation of markers, i.e., JNK and Bax, and the production of ROS in the first possible pathway. Injury by toxins may lead to dysfunctional mitochondria in the second possible pathway. The exposure of pathogens to increasing pathogen-associated molecular patterns (PAMPs) upregulates the expression of caspase 8, thereby leading to mitochondrial permeability transition (MPT) in the third possible pathway. MPT, in the presence of sufficient amounts of ATP, leads to the activation of cytochrome C and apoptotic peptidase activating factor 1 (Apaf-1), which in turn upregulates the expression of caspase 9, 3, 6, and 7 and thus leads to apoptosis [10]. On the other hands, MTP, in the case of severe ATP depletion, leads to necrosis. While, in the case of renal toxicity, multiple stress factors trigger the production of ROS, H_2_O_2_, lactoperoxidase (LPO), glutathione disulfide (GSSG), and C-reactive proteins (CRPs), which in turn raise the levels of malondialdehyde (MDA), lipid peroxidation products (LPPs), total protein carbonyl content (TPCC), blood urea nitrogen (BUN), nitric oxide (NO), creatinine, and various inflammatory markers, hence causing oxidative stress and inflammation in the first pathway [11]. Nephrotoxic substances in the second pathway cause alternation in the hemodynamics of angiotensin II (AT II), NO, and prostaglandins, which in turn decreases the glomerular filtration rate (GFR) and perirenal fat (PRF) and cause cellular damage as shown in Figure 1. Hyperfusion, in the third pathway, also causes injury to epithelial cells, which destroys the cellular membrane. This leads to the activation of caspase 9 and bcl-2, which activates processes such as apoptosis and necrosis and causes cell death [12].

The majority of the synthetic medications used to treat liver and kidney problems are ineffective and can have major negative effects in some cases. The greatest cause of abrupt liver failure in the US is liver toxicity, which has been related to more than 900 medicines [13]. Acetaminophen (APAP), commonly referred to as paracetamol, has been marketed as an over-the-counter painkiller and fever reducer since the 1950s. APAP has long been known to be potentially lethal due to dose-related hepatic and renal impairment [14]. The typical example of direct liver injury is APAP hepatotoxicity. Overdosing on APAP causes nephrotoxicity, which affects about 1–2% of patients [15]. Likewise, severe hepatic and renal damage are common side effects of methotrexate (MTX), a frequently used cytotoxic chemotherapy drug [16]. It is known to inhibit both enzymatic and non-enzymatic antioxidants, thereby increasing oxidative stress in the liver and kidneys [17].

Similar results have been shown with the well-known platinum-containing anticancer drug cisplatin (CP), which has been proven to be quite efficient against a variety of cancers. However, the adverse effects, including nephrotoxicity, hepatotoxicity, gastrointestinal toxicity, and other allergic reactions, restrict the clinical value of CP [18]. The most severe and potentially fatal side effect of CP treatment is nephrotoxicity. The kidneys, in particular the renal proximal tubules, are severely impacted by CP as they are the primary platinum excretory organ [19]. Moreover, doxorubicin (DOX) has been utilized as a first-line anticancer medication in the treatment of solid and hematological malignancies with great effectiveness [20]. Unfortunately, because of its multi-organ toxic effects, particularly those on the liver and kidneys, DOX use has been restricted [21,22]. DOX has been reported to cause organ toxicity via activation of the inflammatory cascade, leading to programmed cell death and apoptosis [23,24]. Thus, there is an urgent need to develop therapies and drugs that have higher efficacy and are safer for the human body in terms of toxicity.

## 2. Conventional Treatments and the Nanotechnology-Based Approaches against Hepato-Renal Damages

Liver and kidney diseases represent a global health issue. Since hepatitis is the most common cause, patients with hepatitis are generally managed with anti-viral treatments. However, in severe cases, liver transplant is needed. Commonly employed drugs approved by the US Food and Drug Administration (FDA) for hepatitis include interferon, tenofovir, and entecavir, which may be prescribed individually or in combination. Lactic acidosis, jaundice, fluid retention, bone problems, anorexia, hypothyroidism, and blood disorders are some other serious side effects which make their use limited [25]. Another degenerative disorder, cirrhosis, has been regarded as an end-stage liver disease that invariably leads to death, unless a liver transplant is performed [26]. Similarly, chronic kidney diseases such as diabetes, hypertension, and glomerulonephritis cause kidney function to deteriorate. Dialysis can help slow the progression of chronic kidney disease to a certain extent, but other treatments are limited, leaving kidney transplantation as the only option [27]. Immunosuppressive medications such as cyclosporine, sirolimus, corticosteroids, and azathioprine are used to prevent organ rejection in patients but can have serious adverse effects such as nephrotoxicity, diabetes, hypertension, increased infection risk, blood clots, seizures, and cardiovascular problems [28].

In this regard, because of their high efficacy and safety, nanoparticles (NPs) have received interest from scientists in recent years [29]. Currently, nanotechnology-based therapeutic and diagnostic approaches have showed enormous promise in improving hepato-renal damage caused by various agents [30]. It has also been shown that the use of NPs for medicinal reasons reduces resistance, removing the main barrier to conventional treatment. Numerous studies are being conducted to find treatments based on nanotechnology that are more accurate and have fewer negative effects than conventional treatments [31]. Nanomedicine, as a translational science, aims to develop low-cost new medicines and diagnostics by leveraging nanotechnology’s enabling capabilities in medicine. Due to their uses in medicine, biology, and material science, metal nanoparticles have stimulated the interest of scientific researchers. Noble metals such as gold and silver offer a lot of potential in biomedical applications, not only for delivering pharmaceutics but also for developing new diagnostic and therapeutic agents [32,33].

Since liver and kidney toxicity is a global problem and conventional treatments for liver and kidney problems are unsuccessful, it is necessary to look for alternative therapies to treat liver and kidney diseases [34]. To date, there has been no satisfactory treatment for significant liver and kidney illnesses, thus the quest for a good hepato-renal protective medicine goes on. As a result, the therapeutic potential of NPs in the treatment of liver and kidney injury is investigated thoroughly in this review article.

## 3. Nanoparticles and Their Interaction with Liver and Kidney Cells

**Figure 2:**
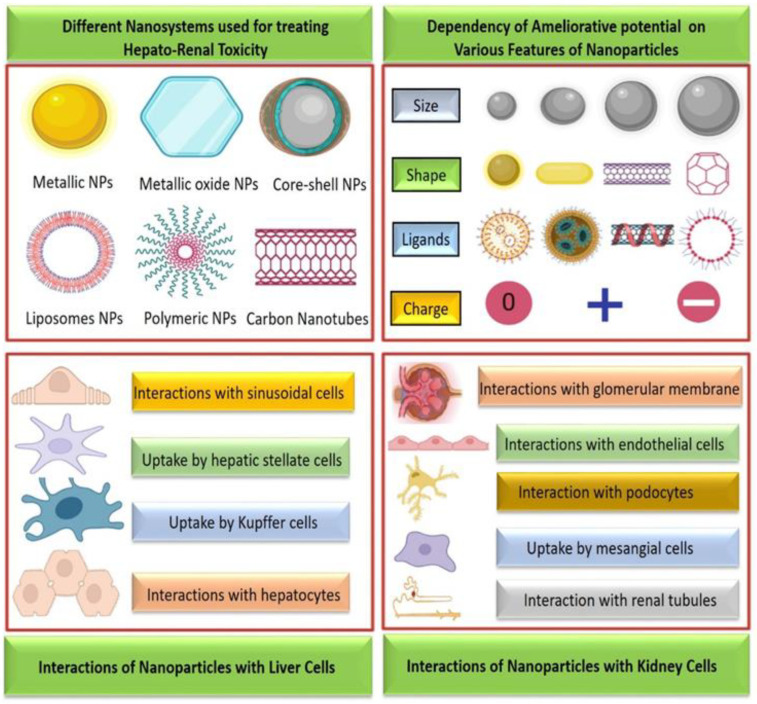
Graphical representation of various nanosystems used for treating hepato-renal toxicity and their interaction with liver and kidney cells.

Due to their features and numerous potential applications, NPs and nanostructured materials play a significant role in nano-biomedical technology [35,36]. The morphological characteristics of NPs can impact their target and circulation throughout the body [37]. Drugs can be encapsulated in nanomaterials or combined with therapeutic drugs for precise distribution to target tissues with a controlled release [38]. Its ameliorative potential, however, is dependent on the size, shape, ligands, and charge (Figure 2). One of the most crucial elements influencing NP characteristics is size. Some optical characteristics of NPs are also influenced by their size. In the UV-visible range, noble metal nanoparticles (NPs) exhibit a powerful extinction band that is absent from the bulk metal spectra [39]. Their form and structure are additional important components. It is possible to purposefully tailor their features by affecting these attributes. The size and shape of NPs are related to the surface charge and it controls stability, aggregation, functional group affinity, and colloidal behavior [40].

The potential of NPs can be completely exploited through their functionalization and modification. This enables management of their interactions with the environment, biocompatibility, colloidal stability, and dispersion [41]. By adding a chemical functional group to the surface, surface functionalization promotes NP self-organization and compatibility. For the required application, this enables the creation of properties such as surface charge and energy, topology, and bioreactivity [42]. Most frequently, in situ synthesis is used to functionalize the surface, which is then further modified by utilizing ligands such as inorganic substances, polymers, biomolecules, and surfactants. Electrostatic, covalent, and non-covalent interactions and intrinsic surface engineering are among the synthetic techniques that enable surface functionalization [41]. These techniques can thus be used to increase the biocompatibility, dispersibility, reactivity, binding capacity, and catalytic activity [43,44].

Hepatocyte cells and non-parenchymal cell types such as sinusoidal endothelial cells (SECs), hepatic stellate cells (HSCs), and Kupffer cells constitute the parenchyma of the liver. The sinusoidal blood capillary barriers are where NPs most likely initially make contact and receive the greatest exposure, supported by the unidirectional hepatic blood flow from the hepatic portal vein/hepatic artery. Most of them are phagocytosed by Kupffer cells before entering SECs but enough may avoid this for SEC internalization [45,46]. Diffusion through their fenestrae or active endocytosis are the two methods used to introduce NP into SECs. Fenestrae line the sinusoidal pole of SEC structures (50-200 nm in diameter) that function as filters to prevent the entry of excessively large NPs or NP complexes (>200 nm) into the liver and divert them towards the spleen [47]. NPs subsequently go through SEC cells to the interior of the liver, where they interact with hepatocytes and HSCs. Accordingly, the four main cell types in the liver are hepatocytes, Kupffer cells, SECs, and HSCs. As a result, each kind of cell is highly specialized and reacts to NPs distinctively [48].

The initial part of the glomerular filtration barrier is composed of glomerular endothelial cells (GECs), which are situated in the glomerular capillary wall. Filtering of the plasma component is greatly aided by GECs and the glycocalyx on their surface [49]. Endothelial filtration problems are linked to renal failure, proteinuria, and other diseases [50]. The glomerular basement membrane (GBM), a particular extracellular matrix connected to GECs, creates a second glomerular filtration barrier. Podocytes are the last line of defense for glomerular filtration and are attached to the GBM’s outside. The glomerular matrix is expanded and balanced by mesangial cells (MCs), which are also in charge of controlling the filtration surface area, removing immunological complexes, and preserving the structural integrity of the glomerular microvascular bed [51]. Drugs used to treat a range of renal disorders in MCs are therefore beneficial [52]. Lastly, the most active cells in renal physiological metabolism are the proximal tubular cells, which actively transport endogenous and exogenous chemicals between the blood and urine. By presenting antigens and releasing cytokines, they can accelerate the tubulointerstitial inflammatory response and the onset and progression of fibrosis [53]. The reduction of tubulointerstitial fibrosis, inflammation, and promotion of renal tubular regeneration are all greatly enhanced by drug administration to the proximal tubular cells [54].

### 3.1. Role of Silver Nanoparticles (AgNPs) in Ameliorating Hepato-Renal Damages

Nano silver exhibits exceptional physical, chemical, and biological properties. It is commonly used to treat wound infections and severe burns. Antimicrobial, antiangiogenic, anticancer, anti-inflammatory, and antioxidant properties of AgNPs have been discovered [55,56,57]. The role of AgNPs in ameliorating hepato-renal toxicity has been displayed in Table 1. In a study, the hepatoprotective potential of AgNPs was assessed against APAP-induced toxicity in a Wistar strain of albino rats. Groups treated with AgNPs reduced the levels of ALT (alanine aminotransferase), AST (aspartate aminotransferase), ALP (alkaline phosphatase), and LDH (lactate dehydrogenase), which were higher in APAP-induced groups. Bilirubin levels were also maintained to normal by treatment with AgNPs, along with restoring the levels of superoxide dismutase (SOD) and catalase (CAT) [58]. Likewise, N-nitrosodiethylamine (NDEA), a strong hepatotoxic agent found in tobacco smoke, water, and a variety of vegetables, has been used to induce hepatotoxicity in male albino rats. Results showed that AgNPs synthesized from leaf extract of *Morus multicaulis* L. significantly ameliorated hepatotoxicity at a dose of 100 µg/kg, with the suppression of IL-6 and IL-10 markers and decrease in the activity of glutamic oxaloacetic transaminase (GOT), glutamic pyruvic transaminase (GPT), and ALP while elevation in the levels of SOD, CAT, and reduced glutathione (GSH) was observed [59]. Moreover, AgNPs also exhibited significant cytotoxic potential in the HepG2 cell line in a dose-dependent manner along with increasing the expression of caspase 3, 8, and 9, marking cell death in the caspase-dependent intracellular pathway. Upregulation of pro-apoptotic factors such as Bax was also reported, thereby acting as a potential chemotherapeutic agent in treating hepatocellular carcinoma [60]. Additionally, the protective impact of berberine-coated nano-silver (BBR-AgNPs) was evaluated on APAP-induced hepato-renal damages in a recent study. APAP-induced diabetic rats showed elevated levels of biochemical parameters and lipid peroxidation, whereas antioxidant levels were found to decline significantly. Administration of BBR-AgNPs ameliorated the damages, thereby exerting a potential antioxidant and anti-diabetic impact [61].

CCl_4_ is one of the most well-known and widely used solvents for inducing liver and renal injuries in laboratory animal models [62]. CCl_4_ is harmful to the kidneys, lungs, brain, heart, and testicles in addition to causing liver damage [63,64]. It produces acute or chronic nephrotoxicity depending on the dose and duration of the exposure [63]. A recent study was conducted to induce hepatotoxicity using CCl_4_ as a toxin. Biochemical analysis showed restoration of LDH and malondialdehyde (MDA) levels to normal, which is an important indicator in the case of tissue injuries in treatment with AgNPs [65]. Likewise, AgNPs showed hepatoprotective activity against CCl_4_-induced toxicity. Low doses (25 mg/kg body weight) of AgNPs were found to be effective in the revival of all the liver parameters without causing toxic effects [66]. Similarly, AgNPs were effective against CCl_4_-induced toxicity in albino rats even at low concentrations and also showed a cytotoxic effect on the HepG2 cell line with great antioxidant and antimicrobial potential [67]. In addition, AgNPs also showed hepatoprotective effects by increasing total protein levels as compared to CCl_4_-treated rats. The liver function enzymes ALT, AST, ALP, and bilirubin were also decreased in groups treated with AgNPs [68].

AgNPs derived from plants and their extracts have been shown to possess antimicrobial activity, anti-inflammatory, and hepatoprotective activity [66]. The pharmacokinetics behavior of AgNPs demonstrates that due to their small size, they may interact effectively with the biological system and can be synthesized in a variety of ways, with green synthesis being the most cost-effective and ecologically friendly among them [69,70,71]. AgNPs synthesized from leaves of aloe vera showed restoration of ALT, AST, and ALP to normal levels. Glucose levels were also significantly reduced in streptozotocin (STZ)-induced diabetic rats [72]. The effect of *Hibiscus rosa-sinensis*-derived AgNPs was evaluated in various liver malignancy cell lines. MTT assay showed a reduction in the viability of cell lines, i.e., pleomorphic hepatocellular carcinoma (SNU-387), hepatic ductal carcinoma (LMH/2A), Morris hepatoma (McA-RH7777), and Novikoff hepatoma (N1-S1 Fudr) in a dose-dependent manner, hence suggesting the ameliorative potential of AgNPs against liver malignancies [73]. Likewise, the protective effects of AgNPs synthesized from *Rhodiola imbricata* were evaluated on the HepG2 cell line. Results showed increased cytotoxicity in a dose-dependent manner, which might be attributed to increased generation of ROS, disrupted respiratory chain of mitochondria, and decreased cellular content of ATP. AgNPs also showed an increase in antioxidant activities in contrast with the control group [74]. Similarly, higher concentrations (10–200 µg/mL) of AgNPs significantly decreased the viability by up to 10%. Apoptosis via the p53 and caspase pathway was triggered in the HepG2 cell line along with ROS generation [75]. Likewise, AgNPs at doses of 100 and 150 µg/kg significantly restored the levels of urea and creatinine, attenuating acetaminophen-induced toxicity in albino rats [76]. Moreover, *Urtica dioica*-derived AgNPs were found to significantly decrease the levels of regucalcin in the treated groups, which is a potent biomarker for the detection of liver injuries [77]. Methotrexate-induced renal damage in rabbits was significantly ameliorated using *Salvia officinalis*-derived AgNPs [78]. Additionally, blood glucose levels were markedly reduced in alloxan-induced diabetic rats using *Solanum nigrum*-mediated AgNPs [79]. Furthermore, AgNPs synthesized from *Tinospora cordifolia* were found to significantly restore the altered serum levels of urea, creatine, uric acid, AST, and ALT in potassium bromate-induced toxicity in albino rats [80]. Thus, AgNPs can be potentially employed as therapeutic agents against hepato-renal damages.

**Table 1:** Summary of the ameliorative effects of AgNPs against hepato-renal damages.

Mode of Synthesis	Morphology	Toxicity Inducing Agent	Experimental Model	Administration Route and Dosage	Molecular Markers	References
Physical method	3–5 nm	Acetaminophen	Female albino rats	Orally, 50, 100, and 150 µg/kg once	↓AST, ALT, LDH, bilirubin, TG, and cholesterol ↑SOD and CAT ↑ATPase and G6Pase	[58]
Chemical method	Spherical, 30.71 nm	-	HepG2 cell line	-, 75 µg/mL for 48 h	↑Bax ↑Caspase 3, 8, 9	[60]
Biological method	Spherical and polydispersed, 30–50 nm	APAP	Albino rats	p.o, 75 mg/kg	↓ALT, AST, LDH, albumin, bilirubin, and cholesterol ↓Urea and creatinine ↓LPO ↑SOD, CAT, and ATPase	[61]
Chemical method	Spherical, 13 ± 1 nm	-	HepG2	-, 50, 100, and 200 µg/ml	↑ROS ↑Bax, p53, and caspase 3 ↓Bcl2	[75]
Chemical method	3–5 nm	APAP	Albino rats	Orally, 50, 100, and 150 µg/kg once	↓Serum urea and creatinine ↓Renal lipid peroxidation ↑GSH ↑SOD, CAT, and ATPase	[76]
Biological method	Spherical, 50 nm	N-nitrosodiethylamine	Male albino rats	Orally, 100 µg/kg for 10 weeks (every alternate day)	↓GOT, GPT, and ALP ↓IL-6 and IL-10 ↑GSH, SOD, and CAT	[59]
Biological method	-	CCl_4_	Male Albino –rats	Orally, 3.25 mg/kg for 8 days	↓LDH, MDA ↑GSH and GPx ↓GOT, GPT, and ALP	[65]
Biological method	Spherical, 32.18 nm	CCl_4_	Swiss Albino mice	-, 100, 150, and 200 mg/kg	↓ALT, AST ALP, bilirubin ↑CAT, SOD GPx, GSSH	[67]
Biological method	Spherical, 13–27 nm	CCl_4_	Swiss Albino mice	Orally, 25 mg/kg for 8 days	↑CAT, GPx, GSH ↓Bilirubin, LDH, MDA ↓GOT, GPT, and ALP	[66]
Biological method	-	CCl_4_	Male Albino rats	Orally, 5 and 10 mg/kg for 3 months before and after 2 h of CCl_4_ administration	↓Regucalcin	[77]
Biological method	Spherical, 98.93 nm	CCl_4_	Albino Wistar rats	Orally, 250 and 500 mg/kg for 14 days	↓ALP, AST ALT, and bilirubin ↑Total protein	[68]
Biological method	Spherical, 20–24 nm	STZ	Wistar albino rats	Orally, 10 mg/kg for 28 days	↓ALP, AST ALT, and MDA ↑CAT, SOD, and GSH	[72]
Biological method	Spherical, 48.52 nm	CCl_4_	SNU-387, LMH/2A, McA-RH7777, and N1-S1 Fudr	-, 223, 185, 265, and 188 µg/ml	↓DPPH	[73]
Biological method	Spherical, 37–42 nm	-	HepG2	-, 200 µg/ml	↓DPPH	[74]
Biological method	Spherical, 20–50 nm	Methotrexate	Wistar albino rabbits	Intramuscularly, 150 mg/kg	↓Serum creatinine, urea, and uric acid ↑GSH	[78]
Biological method	Spherical, 4–25 nm	Alloxan	Wistar Albino rats	Orally, 10 mg/kg for 21 days	↓Blood glucose level	[79]

### 3.2. Role of Zinc Oxide Nanoparticles (ZnONPs) in Ameliorating Hepato-Renal Damages

ZnONPs are among the most widely used metal oxide NPs in a variety of sectors and research-based organizations due to their wide range of applications [81]. Due to their small size, they may easily be absorbed in the human body. ZnONPs could be synthesized in bulk, which makes them inexpensive, and since they are less toxic than other metal oxide NPs, they can be used for a variety of medical applications, including antibacterial, anti-diabetic, anti-inflammatory, anti-aging, wound healing, and bio-imaging [82,83,84,85]. Due to their biocompatibility, ZnONPs can be employed in a wide range of therapeutic sectors as they possess antifungal, antimicrobial, antiviral, and anticancer actions [86]. Several inorganic metal oxides, including TiO_2_, CuO, and ZnO, have been synthesized and are still being explored, but ZnONPs are the most exciting of these metal oxides since they are inexpensive, safe, and simple to prepare [87]. In a recent study, the ameliorative potential of green tea-mediated ZnONPs was shown against ochratoxin-A-induced hepatotoxicity and nephrotoxicity in albino rats (Table 2). Ochratoxin A was found to decline feed consumption and weight gain significantly, with raised levels of ALT, AST, and creatinine. ZnONPs was found to reverse the damage caused by ochratoxin A and was also involved in reducing the levels of ALT, AST, and creatinine. Biochemical analysis showed improvement in kidney tissues, with slight congestion in few areas [88].

Dimethylnitrosamine (DMN) is a carcinogenic, mutagenic, and hepatotoxic chemical. In experimental animals, it is widely reported to cause significant liver cell necrosis and death [89]. A study showed the protective effects of ZnONPs (50 mg/kg) against DMN-induced liver injury in rats. ZnONPs reduced lipid peroxidation, oxidative stress, and fibrosis of the liver along with suppression of TNF-α and IL-12, which indicates a reduction in the levels of proinflammatory cytokines. An increase in the levels of reduced glutathione (GSH) and glutathione peroxidase (GPx) was also observed, thereby improving liver and kidney function [90]. Similarly, ZnONPs (50 mg/kg) declined the levels of MDA, hydrogen peroxide (H_2_O_2_), and nitric oxide (NO) in DMN-induced renal toxicity in vivo [91]. Likewise, *Ochradenus arabicus* (OA)-mediated ZnONPs reduced potassium bromate P-induced hepatotoxicity in Swiss albino rats. The overall health of the treated animals was improved profoundly by maintaining the levels of glutathione reductase (GR), GPx, SOD, and CAT and decreasing the levels of gamma glutamyl transferase (GGT), glutamyl S-transferase (GST), and thioredoxin reductase (TR) using OA-derived ZnONPs [92]. Furthermore, hepatotoxicity induced through cadmium chloride (CdCl_2_) was significantly ameliorated using nano ZnO [93].

Thioacetamide (TAA) is a powerful hepatotoxic and hepato-carcinogenic chemical that is used to stimulate hepatic failure and hepatocyte destruction in experimental animal models [94]. TAA causes hepatotoxicity by producing thioacetamide-S-dioxide, an unstable reactive metabolite that stimulates the production of reactive oxygen species (ROS) by binding covalently to macromolecules [95]. As ZnONPs possess various therapeutic properties, they were employed against TAA-induced hepatotoxicity to evaluate their protective effects. ZnONPs significantly lowered oxidative stress and reduced the expression of inflammatory markers (TNF-α and IL-6) and liver enzymes and also helped in returning the antioxidant status back to its normal level [96]. Likewise, *Eclipta prostrata*-derived ZnONPs showed a dose-dependent cytotoxic effect against the HepG2 cell line. DNA fragmentation assays and activation of caspase 3 validated the apoptotic features of ZnONPs at a concentration of 100 mg/mL [97]. Both ZnONPs and leaf extracts of *Geranium wallichianum* were assessed in conjugation against the HepG2 cell line. The cytotoxicity of the produced ZnONPs was assessed using the MTT test, and the results showed that exposure to different ZnONP dosages for 48 h dramatically reduced the metabolic activity of the HepG2 cell line. The metabolic activity steadily decreased as the concentration of ZnONPs increased over time. At a dosage of 1000 µg/mL, the maximum inhibitory potential (71 percent mortality) was reached, and it was discovered that cytotoxicity declined as the concentration lowered. Thus, the anti-cancerous potential of ZnONPs may be the cause of the decrease in the metabolic activity [81].

The most widespread disease in the world is hepatocellular carcinoma (HCC). Many risk factors have been implicated in HCC-related fibrosis and cirrhosis, including prolonged alcohol intake, viral hepatitis, and fatty liver disease. HCC was induced through diethylnitrosamine (DEN) (200 mg/kg body weight) followed by the induction of CCl_4_ (3 mL/kg) for 3 weeks. Administration of ZnONPs opposed oxidative stress and lowered the biochemical parameters (ALT, AST, and GGT), thereby improving the liver pathology. The scavenging activity of free radicals was also shown by ZnONPs along with a reduction in CRP, IL-6, and TNF-α [98]. Likewise, liver cirrhosis induced by CCl_4_ raised the levels of LDH, ALT, AST, and total protein, which were reduced to the normal levels upon administration of ZnONPs [99]. Moreover, toxicity induced through methotrexate elevated the levels of ALP, GGT, and total protein whereas ZnONPs partially ameliorated methotrexate-induced toxicity and also provided prophylactic relief from the immediate effects of methotrexate [100].

CP, a chemotherapy drug used to treat a variety of malignancies, can cause platinum to build up in the kidney, impairing its function. CP-induced nephropathy raised serum creatinine, blood urea nitrogen, and microalbuminuria, all of which are indicators of renal function. These characteristics, on the other hand, were downregulated after ZnONPs treatment. ZnO-NPs prevented CP-induced decreases in renal superoxide dismutase, catalase, and glutathione reductase, and an increase in renal malondialdehyde levels. Furthermore, the fraction of viable cells was greatly increased while the proportion of apoptotic and necrotic cells was significantly reduced in groups treated with ZnONPs [101]. Similarly, STZ therapy led to diabetic nephropathy in male rats, which was demonstrated by an increase in the blood glucose level, renal oxidative stress markers, and glomerular basement membrane thickness. Administration of ZnONPs intraperitoneally for 7 weeks significantly improved the nephropathy and enhanced renal function [102].

**Table 2:** Summary of the ameliorative effects of ZnONPs against hepato-renal damages.

Mode of Synthesis	Morphology	Toxicity Inducing Agent	Experimental Model	Administration Route and Dosage	Molecular Markers	References
Physical method	Hexagonal, <100 nm	DMN	Wistar rats	Orally, 50 mg/kg on each alternate day for 30 days	↓Lipid peroxidation, oxidative stress, and fibrosis ↓ALT, AST, and LDH ↓TNF-α and IL-12 ↑GSH	[90]
Physical method	-	CdCl_2_	Kunming mice	Orally, 50 mg/kg for 7 days	↓ALT, AST. ALP, LDH, and bilirubin ↑GPx, SOD	[93]
Physical method	Spherical, <100 nm	DMN	Wistar rats	Orally, 50 mg/kg on each alternate day for 30 days	↓MDA, H_2_O_2_, and NO ↑GSH, GPx ↓Creatinine ↑Metallothionein	[91]
Physical method	-	CCl_4_	Male rats	Orally, 25 mg/kg for 14 days	↓ALT, AST, ALP, bilirubin, and LDH	[99]
Physical method	-	Methotrexate	Wistar Albino Rats	Orally, 50 mg/kg for 45 days	↓ALT, AST, ALP, GGT ↑Total protein and albumin ↓Creatinine	[100]
Physical method	˂40 nm	Cisplatin	Sprague Dawley rats	i.p, 5 mg/kg for 7–12 days	↓Serum creatinine, BUN, and microalbuminuria ↓MDA ↑SOD, CAT, and GSH ↓Renal TGF-β1 ↓Bax ↑Nrf2, Ho1, and eNOS	[101]
Chemical method	38–54 nm	TAA	Sprague Dawley rats	i.p, 5, 7.5, and 10 mg/kg for 8 weeks (3 times per week)	↑GSH, CAT, and SOD ↓MDA, ALT, AST, and GGT ↓TNF-α and IL-6 ↓Creatinine, urea, and uric acid	[96]
Chemical method	-	CCl_4_ + diethylnitrosamine	Male Wistar rats	i.p, 5 and 10 mg/kg for 8 weeks daily	↓α-fetoprotein, GPC3, and VEGF ↓MDA, NO ↓IL-6 and TNF-α ↑Plasma glucose and ATP ↓LDL, bilirubin, plasma cholesterol, and triglycerides ↑HDL	[98]
Biological method	Spherical, rod and triangular, 30–40 nm	Ochratoxin A	Albino rats	Orally, 25 ppb for 30 days	↓ALT and AST ↑Total protein and albumin ↓Creatinine	[88]
Biological method	Spherical and oval, 20 nm	PB	Swiss Albino rats	Orally, 5mg/kg twice a week for a month	↓ALT, AST, ALP, LDH ↓GGT, GST, and TR ↑SOD, CAT, GR, and GPx	[92]
Biological method	Triangle, hexagonal, rod and rectangle, 29 ± 1.3 nm	-	HepG2	-, 1–500 µg/ml	↑Cell necrosis ↑ROS ↑Caspase 3,8,9 ↑p53	[97]
-	<100 nm	STZ	Albino rats	i.p, 2.5 mg/kg for 7 weeks	↓Blood glucose, BUN, and MDA ↑SOD, CAT, and GPx	[102]

### 3.3. Role of Gold Nanoparticles (AuNPs) in Ameliorating Hepato-Renal Damages

Due to their promising qualities such as biocompatibility, chemical stability, ease of production, and easy surface modification, gold nanoparticles (AuNPs) are among the most studied metal nanoparticles. Because of their distinctive qualities, AuNPs are used in a variety of medical applications, such as biosensing, medicine delivery, and diagnostics [103,104,105,106]. APAP is being used as an over-the-counter product for pain relief and fever. Excess doses have been reported to cause hepato-renal injury. The effectiveness in preventing acetaminophen-induced toxicity was investigated in a study. AST, ALT, LDH, cholesterol, albumin, and bilirubin levels were significantly elevated in the groups that received APAP; however, therapy with AgNPs returned these levels to normal while also lowering urea and creatinine levels [107]. Likewise, AuNPs synthesized from aqueous bark extract of *Terminalia arjuna* showed excellent therapeutic effects against acetaminophen-induced hepatotoxicity. AuNPs at a dose of 175 µg/kg/day showed restoration of antioxidants, i.e., SOD, CAT, and GSH and reduced levels of MDA, ALT, and bilirubin [30]. Likewise, green-synthesized AuNPs derived from *Terminalia arjuna* showed significant recovery in the expression of inflammatory biomarkers, including kidney injury molecule (KIM-1) expression, against acetaminophen-induced cytotoxicity in a rat model [108]. Many studies reported the ameliorative potential of AuNPs against hepato-renal toxicity as shown in Table 3. 

In another study, the protective effects of silymarin-coated AuNPs were evaluated against cholestasis, a condition characterized by the accumulation of bile acids. Silymarin is widely employed as an efficient hepatoprotective agent. A combination of silymarin-coated nanoparticles showed beneficial effects against oxidative stress, fibrosis, and hepatic cytolysis, thus paving new pathways for the treatment of hepatic damages [109]. Similarly, AuNPs synthesized from *Trigonella Foenum-Graecum* seed extract were evaluated for hepatoprotective effects in male albino mice against CCl_4_ induction. The results showed the intriguing potential of AuNPs, decreasing biochemical parameters (AST, ALT) and proinflammatory markers significantly. Damages were partially recovered using AuNPs as assessed by liver histology [110].

Among the chemicals that are commonly co-administered with illicit narcotics, ethanol is by far the most common, contributing to an increase in hospital admissions and deaths. To increase and prolong the effects of psychostimulant drugs such as methamphetamine (METH) or cocaine, ethanol is regularly combined with them [111,112]. However, the combination of ethanol and METH causes a number of histological changes in the liver. For this, a study aimed to evaluate the lethal effects of ethanol and METH in contributing to liver injury and the use of AuNPs as protective agents. Results showed that treatment with AuNPs (724.96 µg/kg) reduced fibrosis, necrosis, and hepatic cord degeneration. Moreover, a reduction in biochemical markers of liver damage was observed along with a decline in the levels of oxidative stress and proinflammatory cytokines as compared to the injured group [113]. Likewise, the efficacy of silymarin-coated AuNPs was evaluated against CCl_4_-induced liver injury in Wistar rats. A reduction in serum enzymes (ALT, AST, ALP) and alpha SMA and Kupffer cells was also observed. Hepatic stellate cells were inactivated along with enhancement of the hepatic regenerative capacity without inducing side effects on the histological structure of the kidney and lungs [114]. In another study, the potential role of hesperetin-conjugated pegylated AuNPs was evaluated against DEN-induced hepatocellular carcinoma in male albino rats. The findings showed protection of the antioxidant status and a reduction in increased liver parameters, thereby showing anticancerous activity against liver carcinoma [115].

AuNPs synthesized from the seed coat of *Cajanus cajan* are thought to have therapeutic effects. Thus, a study was conducted to evaluate the anticancer effects of *C. cajan* AuNPs against the HepG2 cell line. The investigation demonstrated the production of excessive ROS, which is thought to play a dominant role, inducing apoptosis in cancer cells, thereby enhancing the therapeutic efficiency with minimal side effects [116]. Additionally, AuNPs synthesized from *Cassia fistula* showed promising effects in the treatment of hyperglycemia. A significant decrease in serum biochemistry parameters and an increase in total protein levels and HDL was observed in groups treated with AuNPs against STZ-induced diabetic rats [117].

**Table 3:** Summary of the ameliorative effects of AuNPs against hepato-renal damages.

Mode of Synthesis	Morphology	Toxicity Inducing Agent	Experimental Model	Administration Route and Dosage	Molecular Markers	References
Physical method	-	APAP	Wistar rats	Orally, 50, 100, and 150 µg/kg once	↓ALT, AST, ALP, LDH cholesterol and bilirubin ↓Creatinine and urea ↑GSH, SOD, CAT ↑ATPase and glucose-6-phosphatase	[107]
Chemical method	Spherical, 10 nm	-	Wistar rats	Orally, 0.5 mg for 7 days	↓AST and ALT ↓MDA ↓TGF-β1	[109]
Chemical method	Spherical, 7.4 ± 1.6 nm	Alcohol-methamphetamine	Wistar rats	Orally, 181.48, 362.48, and 724.96 µg/kg for 28 days	↓MPO and MDA ↑GSH ↓IL-1β and TNF-α ↓ALT, AST, and triglycerides ↓Kupffer and hepatic stellate cells	[113]
Biological method	Spherical, 7–20 nm	APAP	Wistar albino rats	i.p, 55, 175, 550, 2000 µg/kg/day for 14 days	↓GOT, GPT, ALT, bilirubin, and MDA ↑SOD, CAT and GSH	[30]
Biological method	-	CCl_4_	Swiss albino male mice	Orally, 0.5 mg/kg	↓ALT, AST, ALP, ACP, and total bilirubin ↓IL-1β, IL-17, and TNF-α ↓LPO ↑IL-10 and Cathepsin K ↑GSH, SOD, and CAT	[110]
Biological method	Spherical, 20 nm	CCl_4_	Wistar rats	Intragastrically, 30 mg/kg for 14 weeks	↓Kupffer cells, hepatic stellate cells, and Alpha SMA	[114]
Biological method	-	DEN	Wistar albino rats	i.p, 1.5 mg/0.5 mL, twice a week for 16 weeks	↓LPO and lipid hydroperoxides ↓AST, ALT, ALP, LDH, and gamma GT ↑SOD, CAT, GSH, GPx, and GR ↑ATPase, Na^+^/K^+^ ATPase, Mg^2+^ ATPase, and Ca^2+^ ATPase	[115]
Biological method	Spherical, 29 nm	-	HepG2	-, 2, 4, 6, 8, 10 µg/mL	↑Apoptosis	[116]
Biological method	-	APAP	Wistar albino rats	i.p, 175 µg/kg/day for 14 days	↑IL-10 ↓KIM-1, Cystatin C, TNF-α, and IL-18	[108]
Biological method	Spherical, 55.2–98.4 nm	STZ	Albino wistar rats	Intragastrically, 60 mg/kg for 30 days	↓Serum glucose levels ↓HbA_1C_ ↓AST, ALT, and ALP ↑Albumin, globulin, and total protein ↓Serum urea, creatinine, and uric acid ↓Total cholesterol, triglycerides, and LDL ↑HDL	[117]

### 3.4. Role of Other Nanoparticles in Ameliorating Hepato-Renal Damages

Aluminum is the third most prevalent metallic element on the planet, accounting for around 8% of all mineral components in the crust. Aluminum’s biotoxicity has received more attention recently as a result of its widespread availability [118]. Since humans are particularly vulnerable, hepato-renal toxicity may occur from the accumulation of aluminum in the liver and kidneys [119]. Selenium (Se) is a critical micronutrient for human health; in addition to its presence in oxido-reductase selenoenzymes, it has anti-carcinogenic, anti-muscular dystrophy, anti-aging, and antioxidant properties [120,121,122]. Therefore, a study was conducted to assess the ability of selenium nanoparticles (SeNPs) to mitigate the hepatorenal toxicity caused by aluminum chloride in albino rats. According to the findings, SeNPs were significantly able to repair the harm that was brought on by elevated liver and renal function parameters. SeNPs also decreased hepatic and renal MDA contents along with the increase in all antioxidative parameters. The structural integrity of the liver was also maintained, which prevents the leakage of hepatic enzymes (Table 4) [123]. Likewise, starch-based SeNPs were evaluated against melamine-induced hepatorenal toxicity in albino rats. Interestingly, administration of starch-based SeNPs resulted in remarkable protection in rats treated with melamine through the quenching of oxidative stress and increase in antioxidant parameters [124]. Moreover, SeNPs against APAP-induced hepatic damage possessed significant capability to restore the cellular structure and avoided further damage, thereby presenting a novel strategy for employing SeNPs as a hepatoprotective agent in the field of medicine [125]. Additionally, STZ-induced diabetes mellitus showed severe biochemical and histological changes in the architecture of the liver and kidney, wherein SeNPs have protective effects and reduced the risk of diabetes complications [126].

Acute kidney injury (AKI), which has a high mortality and morbidity rate worldwide, is a significant public health concern. Hypoxia, ischemia, and exposure to nephrotoxic chemicals are the main contributors [127]. An established experimental paradigm for investigating and comprehending the underlying biochemical and molecular mechanisms of AKI is glycerol-induced AKI [128]. Renal vasoconstriction, tubular necrosis, and myoglobinuria are all symptoms of glycerol-induced nephrotoxicity in rats, just like in humans [129]. A study aimed to evaluate the potential effects of SeNPs against glycerol-induced nephrotoxicity in albino rats. SeNPs were found to alleviate kidney function parameters, i.e., serum urea and creatinine, and were also found to inhibit oxidative stress by restoring thee antioxidant balance along with the suppression of proinflammatory cytokines, thereby depicting the anti-inflammatory, anti-apoptotic, and antioxidant potential of SeNPs [130].

Chitosan has received a lot of interest as a biomedical material because of its wide range of biological activities, including anticancer, immune-stimulating, anti-allergic, anti-coagulant, and anti-inflammatory properties [131,132,133]. However, chitosan’s applicability is severely limited due to its large molecular weight and water insolubility. Nanoparticle formulation as a treatment is the biological basis for improving the therapeutic response and oral absorption of chitosan and other drugs that are not easily soluble [134]. It has been demonstrated that chitosan nanoparticles (CNPs) offer chitosan better immune-enhancing, antibacterial, and anticancer characteristics [135]. Significant hepatoprotection by CNPs was shown in a dose-dependent fashion in CCl_4_-induced hepatotoxicity. Antioxidant parameters were also boosted, with reversal in the damage to the liver architecture, indicating therapeutic efficiency in liver disorders as discussed in Table 4 [136].

Cerium oxide nanoparticles (CeO_2_NPs) are well known to reduce the levels of ROS and inflammatory mediators, i.e., TNF-α, iNOS, and interleukins in vitro [137]. As a result, speculation has increased that CeO_2_NPs could be effective in the prevention and/or treatment of diabetic cardiomyopathy, lung illness, retinal degeneration, stroke, and neurodegenerative disorders [138]. Thus, a study aimed to evaluate the protective effects of CeO_2_NPs against hepatic damage. Findings demonstrated raised levels of serum biochemical parameters in CCl_4_-treated rats, which were improved upon the administration of CeO_2_NPs. The expression of genes related to proinflammatory cytokines and cell differentiation was also reduced. In addition, systemic signs of reduced liver inflammation were also observed as evidenced by decreased levels of AST and ALT enzymes [139].

The hepatic and renal systems are affected by lead (Pb) intoxication, resulting in a homeostasis imbalance. Curcumin is a powerful antioxidant; however, due to its low bioavailability, it has limited clinical applications. The use of cockle shell-derived aragonite calcium carbonate nanoparticles (CSCaCO_3_NP) to improve the effectiveness and targeted distribution of curcumin has been proposed as a promising prospect in nanomedicine (Cur). A study aimed to investigate whether curcumin-loaded CSCaCO_3_NP (Cur-CSCaCO_3_NP) could ameliorate rats with lead-induced hepato-renal damage. Oral administration of Cur-CSCaCO_3_NP (50 and 100mg/kg bw) showed a significant decline in the levels of liver and kidney function parameters and was also observed to increase the levels of antioxidants, hence attenuating oxidative stress. Thus, Cur-CSCaCO_3_NP showed better therapeutic effects in comparison with free curcumin and could be employed as a novel approach to treat Pb-induced liver and renal impairments [140].

**Table 4:** Summary of the ameliorative effects of other NPs against hepato-renal damages.

Nanoparticles	Mode of Synthesis	Morphology	Toxicity Inducing Agent	Experimental Model	Administration Route and Dosage	Molecular Markers	References
SeNPs	Chemical method	-	AlCl_3_	Albino rats	Orally, 0.4 mg/kg for 21 days	↓AST, ALT, ALP LDH, total bilirubin ↓Creatinine, urea, and uric acid ↓MDA ↑GSH, SOD, GPx	[123]
SeNPs	Chemical method	Spherical, 10–20 nm	APAP	Sprague Dawley rats	i.p, 0.5 mg/kg for 2 times once	↓ALT, AST, and ALP ↓MDA ↑CAT, SOD, GR, GSH	[125]
SeNPs	Chemical method	Spherical, 19 ± 1 nm	STZ	Albino rats	Orally, 0.1 mg/kg for 28 days	↓Blood glucose levels ↓Total lipid, total cholesterol, triglyceride, LDL ↑Glucose-6-phosphated dehydrogenase activity ↑HDL	[126]
SeNPs	Chemical method	105.5 nm	Glycerol	Wistar albino rats	Orally, 0.1 mg/kg for 14 days	↓Serum rea, creatinine ↓Kim-1, MDA, NO ↓TNF-α, IL-1β, cytochrome c, Bax, and caspase 3 ↑GPx, GR, SOD, and CAT	[130]
SeNPs	Starch stabilized SeNPs	Spherical, 20–140 nm	Melamine	Albino rats	Orally, for 28 days	↓ALT and AST ↓Serum urea and creatinine ↓MDA ↑GSH	[124]
Chitosan NPs	-	Rod, 100 nm	CCl_4_	Sprague Dawley rats	Orally, 140 and 280 mg/kg	↓ALT, AST, ALP, CEA, and AFP ↓MDA ↑GPx, SOD, and CAT	[136]
CeO_2_NPs	Chemical method	Spherical, 4–20 nm	CCl_4_	Albino rats	Orally, 0.1 mg/kg twice weekly for 2 weeks	↑Albumin and total protein ↓AST and GGT ↓Caspase 3 ↓IL-1β, TNF-α, iNOS and COX-2 ↓ROS	[139]
Cur-CSCaCO_3_NPs	-	-	Pb	Sprague Dawley rats	Orally, 50 and 100 mg/kg three times a week for 8 weeks	↓ALP, AST, ALT, total bilirubin, and LDH ↑Total protein ↓Serum urea and creatinine ↑SOD ↓MDA	[140]

## 4. Possible Mechanisms Involved in Ameliorating Hepato-Renal Damages by NPs

**Figure 3:**
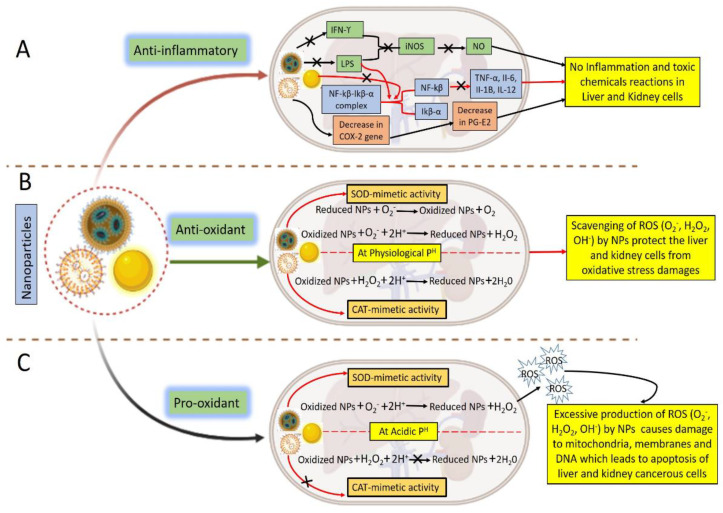
Possible mechanism adopted by NPs to ameliorate the hepato-renal toxicity: (**A**) Anti-inflammatory; (**B**) antioxidant; and (**C**) pro-oxidant.

NPs have demonstrated an ability to reduce hepato-renal toxicity via three primary mechanisms, i.e., by acting as an anti-inflammatory agent, an antioxidant, and a pro-oxidant. In the case of anti-inflammatory action, the generation of high nitric oxide (NO) enhances the expression of inducible nitric oxide synthase (iNOS), which is harmful to cells. NO regulates host immune cell activity, which in turn regulates local immunity and destroys nearby tissues, making it one of the primary causes of inflammation. Unsafe chemical reactions can occur in other host tissues as a result of the excessive NO generation that occurs during some types of inflammation. IFN-γ and LPS significantly increased iNOS expression, which was significantly decreased when NPs were administered to the therapy. In a dose-dependent way, NPs have been shown to decrease NO generation by IFN- γ plus LPS-activated macrophages as shown in Figure 3 [141,142]. Proinflammatory cytokines, including IL-6, IL-1β, IL-12, and TNF-α, are principally responsible for causing inflammation. These cytokines are responsible for the activation of inflammatory reactions. They are known to promote mast cell growth and expansion. Active nuclear factor kappa B (NF-kB) promotes the inflammatory response by enhancing the expression of specific genes that maintain cell proliferation [143,144]. Pro-IL-1β is converted from an inactive to an active state by an enzyme known as caspase-1. In activated mast cells, NPs prevent both NF-kβ and the caspase-1 enzyme from functioning. In addition, IkB-α, an inhibitory protein, forms a compound with NF-kB while it is inactive. Proinflammatory mediators such as LPS aid in the release of NF-kB from this complex by prompting IkB-α to phosphorylate. IkB-α is phosphorylated, which impairs its capacity to bind to NF-kB. The phosphorylation of IkB-α is known to be inhibited by NPs, blocking the release of NF-kB. NPs also prevent the expression of COX-2 and iNOS. Additionally, NPs prevent LPS from activating COX-2 in macrophage cells, which prevents the release of PG-E2 as represented in Figure 3A [145]. Through this anti-inflammatory action, medical conditions, i.e., high blood pressure, glomerulonephritis, acute kidney injury, and amyloidosis, have been found to be reduced by administration of NPs [76,146,147,148].

NPs are also known to act as an antioxidant as depicted in Figure 3B. By entering the cell through endocytosis and scavenging ROS (O2-, H_2_O_2_, or OH) due to SOD mimetic activity in which superoxide is reduced into H_2_O_2_ and CAT mimetic activity in which H_2_O_2_ is further degraded into water, NPs protect normal cells by acting as antioxidants in cells at physiological pH, thus preventing conditions such as cirrhosis, hepatitis, necrosis, and acidosis [149,150,151].

NPs can also enter cancer cells by receptor-mediated endocytosis in the case of pro-oxidant activity as illustrated in Figure 3C. The acidic intracellular pH of cancerous cells stimulates the NPs to mimic SOD-like activity, which reduces superoxide into H_2_O_2_ but inhibits CAT-mimetic activity, causing a considerable amount of H_2_O_2_ to be generated in the cancer cell. This is because superoxide is reduced by SOD into H_2_O_2_. These ROS also harm mitochondria, oxidize proteins, and denature DNA, which results in the demise of cancer cells [152,153]. This pro-oxidant action has also been found to prevent renal cysts, acute nephropathy, renal hypouricemia, cancers, and diabetes [154,155,156,157].

## 5. Conclusions and Future Recommendations

Although there have been considerable improvements in diagnosis, toxicities related to the liver and kidney are still the top cause of death globally. There is currently no effective treatment for hepato-renal damages, and all conventional therapies and medications have deleterious side effects. Investigators are therefore endeavoring to develop better treatment techniques that have a tendency to possess higher specificity, efficacy, and low toxicity. Nanotechnology has spurred hope that life-threatening illnesses related to the liver and kidney will be effectively treated in the near future during this time of despair. NPs have thus emerged as an ideal candidate due to their longer retention duration, adaptable shape, and slower rate of agglomeration. NPs are a great option for treating hepato-renal impairments because of their anti-inflammatory, antioxidant, and pro-oxidant mechanisms. As the impact of NPs can vary depending on the disease condition, its cellular response, however, can be crucial. More research is still required to determine the precise molecular mechanism by which NPs act to ameliorate adverse reactions to the organs upon administration of hazardous substances.

## Data Availability

All the data are included in this study.
